# Situational self-assertion in self–other dilemmas among young adults in five cultures: negotiations between self and others

**DOI:** 10.3389/fpsyg.2026.1766805

**Published:** 2026-03-03

**Authors:** Mika Hirai, Susie D. Lamborn

**Affiliations:** 1 Department of Psychology, University of the Sacred Heart, Tokyo, Japan; 2 Educational Psychology Department, University of Wisconsin–Milwaukee, Milwaukee, WI, United States

**Keywords:** autonomy, relatedness, self-assertion, self–other dilemmas, young adults, culture

## Abstract

Autonomy and relatedness are essential and universal human needs that are manifested in different cultures with some variations. This study examined situational self-assertion as a behavioral expression in self–other dilemmas among young adults in five different cultures: the United States, China, Korea, Taiwan, and Japan. Four hypothetical self–other dilemma stories were designed to vary in Importance (Low/High) and participants responded to each scenario with respect to Type of Other (Parent/Friend). Participants were asked to answer what they would do in these situations, reflecting whether they would prioritize their own needs or those of others. Results indicated self-assertion varied systematically depending on the importance of the situation and who the target other was; more specifically, young adults across cultures were more self-assertive for situations that were highly important to themselves. At the same time, culturally differentiated patterns emerged in how self-assertion was calibrated toward parents and friends. Taken together, these findings highlight self-assertion as a context-sensitive behavioral process through which autonomy and relatedness are jointly negotiated in self-development, rather than as a fixed cultural disposition or a zero-sum trade-off between the two.

## 1 Introduction

Human development is grounded in two fundamental and sometimes competing tendencies: the striving for autonomy and the need for relatedness (Bakan, 1966; Erikson, 1950). From infancy onward, individuals rely on caregivers for safety, socialization, and emotional support, while simultaneously pursuing their own independent needs and interests. Developmental theories, including attachment theory, have emphasized that exploration, competence, and self-confidence emerge within the context of close relationships, highlighting that autonomy and relatedness are not mutually exclusive but rather coexist as parallel developmental imperatives. The present study focuses on self-assertion as a situational behavioral expression through which individuals negotiate autonomy and relatedness in concrete interpersonal dilemmas.

During adolescence and young adulthood, the balance between autonomy and relatedness becomes especially salient. Developmental research has shown that as adolescents begin to separate more clearly from parents and to form deeper relationships with peers, they encounter an increasing number of situations in which personal needs and social expectations collide (e.g., Collins and Laursen, 1992; Koepke and Denissen, 2012; Montemayor, 1983; Steinberg, 2001; van Rijn–van Gelderen et al., 2025). Recent longitudinal and meta-analytic work further demonstrates that autonomy-related conflicts increase as adolescents renegotiate relational boundaries with parents and simultaneously establish more committed friendships and romantic relationships (Schulz et al., 2023; Wang et al., 2023). (Grotevant and Cooper 1985) showed that adolescents' individuation within families involves balancing individuality and connectedness, with effective negotiation requiring not only self-assertion but also mutuality and permeability. In this context, self-assertion can be understood as a concrete behavioral process through which adolescents articulate their own needs, preferences, or positions when these come into tension with relational expectations (Smetana, 1989, 1995; Smetana and Rote, 2019). Rather than reflecting autonomy or relatedness *per se*, self-assertion represents a situationally enacted strategy that makes the negotiation between these two motivational dimensions visible at the behavioral level. Extending this view, (Lichtwarck–Aschoff et al. 2008, 2009) demonstrated that moderate levels of daily mother–daughter conflict foster emotional variability—flexible self-regulation within relationships—which serves as a precursor to identity development. Together, these studies indicate that self–other conflicts in supportive family climates can play a constructive role in identity formation (Öztürk et al., 2023; van Rijn–van Gelderen et al., 2025). Importantly, such conflicts are not typically pathological; rather, they often serve as developmental opportunities for reorganizing relationships and redefining boundaries (Laursen et al., 1998). Such findings suggest that it is not the presence or absence of conflict, but how adolescents express and negotiate their own positions within relationships, that is developmentally consequential.

Moreover, friendships and other close peer relationships also provide a key context in which adolescents and young adults navigate self–other dilemmas. Conflicts with friends, often involving issues of loyalty, trust, or autonomy, are common yet typically resolved constructively, contributing to social competence and relationship-maintenance skills (Laursen, 1995; Bukowski et al., 2018). Research further shows that adolescents' conflict resolution strategies with friends develop over time and differ across individuals, illustrating that peer conflicts, like those with parents, offer opportunities to learn how to balance autonomy and relatedness in everyday life (Laursen and Collins, 2009; Yu et al., 2014). In sum, young people must maintain a balance of both autonomy and relatedness depending on the situation and kinds of others involved.

One central process in balancing self–other tensions is self-assertion, defined as the capacity to articulate one's needs, preferences, and opinions during social interactions. Self-assertion has been conceptualized as a socially embedded behavioral skill that enables individuals to express their own standpoint while remaining engaged in ongoing relationships (Alberti and Emmons, 2008; Deluty, 1979). Importantly, self-assertion is distinct from both aggression and passivity: whereas aggressive behavior prioritizes the self at the expense of others, and passive behavior involves the suppression of one's own needs, self-assertion involves the regulated expression of the self within interpersonal contexts. Developmental research has highlighted the role of self-assertion in autonomy development, particularly during adolescence and young adulthood. From a self-determination theory perspective, autonomy refers to the experience of acting with a sense of volition and self-endorsement, rather than independence from others *per se* (Ryan and Deci, 2017). Accordingly, self-assertion can be understood as a behavioral mechanism through which autonomy is enacted and negotiated within relational contexts (Soenens and Vansteenkiste, 2010). The present study aimed to examine how young adults in five cultural contexts negotiate self–other dilemmas, thereby illuminating both the cultural commonalities and differences in self-assertions during young adulthood. It is assumed that when dilemma situations place the self and others in conflict, individuals carefully balance between both according to the situational importance of the dilemma and the target person involved (parent vs. friend).

In Western psychological traditions, especially those rooted in humanistic and clinical perspectives, assertiveness has been regarded as a core indicator of autonomy because articulating one's needs supports the development of an agentic and coherent sense of self (e.g., Rogers, 1961; Maslow, 1999). However, cultural frameworks indicate that the meaning, value, and expression of self-assertion vary across societies. For instance, it is assumed that, in non-Western cultural contexts, where people place a high value on attuning to interpersonal relationships and to the situational atmosphere, self-assertion may be more context-dependent, varying with the specific situation and content of what is being asserted.

Accordingly, it is necessary to distinguish between different situations in which self-assertion occurs and examine how culturally shaped patterns of context-dependent self-assertion may differ. Using hypothetical dilemmas and a think-aloud method, (Hirai 2000) and (Hirai and Takahashi 2000) showed that Japanese college students differentiated their responses according to the importance of the issue and the type of target person, prioritizing their own needs in highly important matters (e.g., marriage, career) but yielding more readily in less serious situations. They also tended to assert their own preferences more strongly with family members than with friends or other figures. Subsequent studies with elderly adults and young children revealed similar patterns, with individuals across age groups showing greater self-assertion when issues were personally significant (Hirai, 2006, 2017). These findings indicate that self-assertion is not a fixed characteristic, but a dynamic process shaped by situational and relational contexts.

Understanding how individuals balance autonomy and relatedness has also required attention to cultural contexts. Early theoretical frameworks in cultural psychology, such as individualism vs. collectivism (Triandis, 1995) and independent vs. interdependent self-construals (Markus and Kitayama, 1991), emphasized cultural contrasts in the prioritization of personal independence vs. social connectedness. (Rothbaum et al. 2000) similarly contrasted cultural pathways of development in the United States and Japan as generative tension and symbiotic harmony. These perspectives established autonomy and relatedness as central constructs in the study of cultural variation.

However, these dichotomous models tend to oversimplify cultural dynamics. Many developmental researchers have argued that autonomy and relatedness coexist across cultures in ways that cannot be captured through bipolar assumptions (Keller, 2016, 2017; Raeff, 2006, 2010). This shift toward a more nuanced view is reflected in Self–Determination Theory (SDT; Ryan and Deci, 2017), which posits that autonomy, competence, and relatedness are universal psychological needs. (Vansteenkiste et al. 2020) argue that although the expression of these needs varies across cultural environments, fulfilling them promotes wellbeing globally, and that universality does not imply uniformity; cultural contexts shape the conditions under which autonomy and relatedness are enacted. Consistent with this view, (Beyers et al. 2024) emphasize that the development of autonomy is both a universal and culturally embedded process: while autonomy becomes increasingly salient during adolescence across societies, its expression and supportive contexts vary depending on cultural norms and relational expectations.

A major theoretical contribution that advances this view is Kagitçibaşi's (2005, 2007, 2013) model of the “autonomous–related self.” Her “family model of psychological/emotional interdependence” conceptualizes agency (autonomy–heteronomy) and interpersonal distance (separation–relatedness) as two dimensions that can be used to yield four patterns of self-development. Among these, the autonomous–related self is proposed as the most adaptive in contemporary societies because it integrates personal agency with relational connectedness. Kagitçibaşi argued against the assumption that modernization leads inevitably to independence; instead, she proposed that psychological interdependence—close family ties combined with autonomy—is both viable and advantageous. Recent empirical work supports this perspective. (Liang et al. 2021), for example, developed and validated a four-dimensional measure of parents' developmental goals in the United States and China, demonstrating that parents in both cultures simultaneously value autonomy and relatedness for their children. Their findings reinforce and extend Kagitçibaşi's model by showing that the autonomous–related orientation is not only culturally viable but also increasingly evident across diverse sociocultural contexts. Whereas this line of research focuses on autonomy and relatedness as relatively stable values or developmental orientations, the present study examines how these two needs are behaviorally negotiated in concrete interpersonal situations. From this perspective, examining situational patterns of self-assertion offers a concrete way to investigate how autonomy and relatedness are jointly negotiated in everyday interpersonal contexts without collapsing them into a single dimension.

Rather than directly measuring autonomy and relatedness as motivational constructs, the present study examines how these concerns become behaviorally expressed through self-assertion in specific dilemma situations between self and others. Recent integrative reviews of adolescent development emphasize that autonomy should not be equated with independence or separation from others but rather understood as a sense of volition and self-endorsement that is negotiated within ongoing relationships (Smetana et al., 2024). From this perspective, autonomy and relatedness are not opposing forces but dynamically coexisting processes that are jointly constructed through everyday interactions, including conflict and negotiation with significant others. Importantly, (Smetana et al. 2024) argue that autonomy is best examined not only as an internal motivational state, but also through observable interpersonal processes, such as how adolescents express their own positions, negotiate disagreements, and manage self–other tensions in context. This view provides a strong conceptual foundation for examining self-assertion as a situational behavioral expression through which autonomy and relatedness are jointly enacted.

Importantly, the present study does not treat self-assertion as a direct indicator of either agency or interpersonal distance, nor as a combination of these two dimensions into a single continuum. Rather, self-assertion is conceptualized as a situationally enacted behavioral expression through which individuals articulate their own preferences or positions within ongoing relationships. From this perspective, self-assertive behavior may reflect the expression of agency within relational contexts, but it does not imply low relatedness or psychological separation. Thus, higher or lower levels of self-assertion in this study should not be interpreted as reflecting greater autonomy or greater relatedness *per se*, but rather as variations in how individuals express their own positions within relational and situational contexts.

More recent theoretical developments further strengthen the move beyond dichotomous cultural models. (Kitayama and Salvador 2024) proposed an “eco-cultural complex” framework, arguing that cultural patterns of autonomy and relatedness arise from dynamic interactions among ecological conditions, historical trajectories, and institutional structures. Their broader program of research demonstrates that cultural mindsets are enacted through daily practices and situational affordances (Kitayama et al., 2022), illustrating how autonomy- and relatedness-related processes become psychologically meaningful within culturally structured contexts. These contributions underscore that autonomy and relatedness are culturally organized developmental potentials that take diverse forms across societies.

Despite these integrative perspectives, cultural variation remains pronounced, especially within East Asia. Even within East Asia, parent–child relationships are thought to differ greatly across cultures, largely because the influence of Confucian thought varies from one culture to another. Confucian values emphasize family interdependence, filial duty, and respect for parental authority, but their expression varies across East Asian cultures. For example, (Takahashi et al. 2020) showed that relationships with parents, especially that of fathers, differed markedly between Chinese and Japanese adolescents and young adults, and those differences were partially explained by cultural beliefs that pertained to patriarchal values, which are stronger in China than Japan. Among Korean young people, parental authority also remains strong, and it is well known that children commonly use honorific language when speaking to their parents. It is widely recognized that the influence of Confucianism is much stronger in China and Korea, but relatively weaker in Japan. Although East Asian societies such as Japan, South Korea, China, and Taiwan are frequently described as a single cultural region, recent comparative research reveals meaningful cross-national differences in family values and relational orientations (e.g., Lin and Yi, 2013). Recent research further suggests that gender and family attitudes within East Asia do not follow a simple traditional–modern dichotomy but may instead reflect aligned, competing, or blurred value configurations (Han and Oh, 2025). It is suggested that the balance between autonomy and relatedness in East Asia follows multiple developmental pathways rather than a uniform trajectory across societies.

Important questions remain regarding how culturally organized values and everyday interpersonal contexts jointly shape self-assertion. A central issue is whether the context-sensitive patterns of self-assertion observed among Japanese individuals reflect a culturally distinctive phenomenon or whether similar dynamics are found in other societies. For instance, does the commonly assumed emphasis on individuality in Western cultural contexts imply that people in these societies consistently behave in self-assertive ways across situations? Conversely, do East Asian cultural norms surrounding relational harmony mean that individuals uniformly suppress their own preferences in the interest of maintaining smooth interpersonal relationships? This is unlikely to be the case. Across cultural contexts, individuals are expected to engage in flexible forms of self-assertion depending on the importance of the issue and the nature of the relationship involved. At the same time, however, culture-specific variations in how people calibrate their responses to such situational demands are also likely to emerge, reflecting culturally shared expectations internalized through developmental processes. These considerations suggest that examining self-assertion within specific dilemma situations provides a valuable window into understanding cultural similarities and differences.

Building on these considerations, the present study advances prior work by directly comparing young adults from five cultural contexts to clarify how situational demands and culturally grounded expectations jointly shape self-assertion in self–other dilemmas. We examined cultural commonalities and differences in self-assertiveness using hypothetical dilemma situations in which benefits to the self and others were in conflict. Participants were recruited from one Western country (the United States) and four East Asian societies (China, Korea, Taiwan, and Japan). Following previous research, we focused on two situational factors: the importance of the situation (high vs. low) and the type of interaction partner (parent vs. friend), both of which have been shown to influence decisions about whether to prioritize autonomy or relational considerations. Based on prior theory and empirical findings, we formulated predictions at two levels: (a) general situational patterns of self-assertion expected across cultures, and (b) culturally differentiated patterns reflecting differences between Western and East Asian contexts, as well as variation within East Asia.

Across cultural contexts, we expected individuals to engage in situation-sensitive self-assertion, that is, to prioritize their own needs more strongly in highly important situations than in less important ones, regardless of culture. At the same time, responses were expected to differ depending on the relationship target. In low-importance situations, young adults may prioritize their own needs more readily with parents than with friends, given that friendships are more voluntary and often require greater sensitivity and impression management. In contrast, in high-importance situations such as major life decisions, differences between parents and friends may diminish because individuals may be more likely to assert themselves irrespective of the interaction partner. We therefore anticipated interactive effects between situational importance and relationship type.

We also predicted cultural differences in these patterns. While situationally dependent self-assertion should appear across all societies, the degree and direction of self-assertiveness were expected to vary between Western and East Asian cultures, as well as among East Asian societies themselves. In line with previous work, Americans were expected to show higher overall self-assertiveness than participants from East Asian contexts. Within East Asia, we anticipated lower self-assertion toward parents in societies where Confucian values are more strongly embedded such as China and Korea, particularly in highly important situations, because parental authority and filial obligations are emphasized. In contrast, young adults in Japan and Taiwan, where autonomy-supportive and flexible family norms have become more common, may be more willing to assert themselves even toward parents, especially regarding personal life choices. Cultural differences toward friends were expected to be smaller, as friendships generally follow more egalitarian norms and are less constrained by hierarchical expectations.

In addition, we also explored potential gender differences in self-assertion. Prior research suggests that boys are often encouraged to express autonomy more strongly within families, whereas girls may be socialized toward relational sensitivity. Because such gendered expectations may intersect with cultural and situational demands, we examined gender as a supplementary factor.

## 2 Method

### 2.1 Participants

A total of 1,239 university students in the U.S. (Milwaukee), China (Beijing and Nanjing), Korea (Seoul), Taiwan (Hualien), and Japan (Yokohama) completed questionnaires. Specifically, 147 students in the U.S. (94 females and 53 males, 19–28 years, *M* = 21.71, *SD* = 2.16), 212 students in China (108 females and 104 males, 18–26 years, *M* = 20.35, *SD* = 2.04), 261 students in Korea (162 females and 99 males; 18–28 years, *M* = 21.61, *SD* = 2.30), 195 students in Taiwan (Hualien County, 107 females and 88 males, 18–29 years, *M* = 19.91, *SD* = 2.08), and 424 students in Japan (Yokohama, 282 females and 142 males; 18–27 years, *M* = 19.32, *SD* = 1.25). Students were primarily from middle-class families in all countries.

In all countries, participants were recruited in classes in which their participation was voluntary, and answers were collected anonymously. All participants were informed that they could choose not to participate in the study and there would be no penalty for doing so. Students provided informed consent before completing the questionnaire. This study received ethical approval from the institutional review board (IRB) at the authors' universities.

### 2.2 Self–other dilemmas

A set of four hypothetical stories depicting conflicts between one's own needs and those of others were constructed based on our former studies (Hirai, 2000, 2006; Hirai and Takahashi, 2000) and piloted with the cultural samples represented in the study. Four types of hypothetical self–other dilemma stories were designed, in which participants were asked to answer what they would do in these situations, whether they would prioritize their own needs or those of the others. The four dilemmas involved choosing who to marry, to take a particular job, to eat at a certain restaurant, and which TV show to watch. All the questions were first developed in Japanese and then were translated into four languages by bilinguals and were checked via back–translations. Stories were presented randomly.

#### 2.2.1 Self-assertion

The key dependent variable was the Self-assertion Score. Participants were instructed to think “if I were in this situation” and to answer what their own reaction would be. Answers to four items were averaged to construct a Self-assertion Score. Participants answered 4 items on a 6–point Likert scale (from 1 = “Strongly disagree” to 6 = “Strongly agree”). The items prepared for each scenario were constructed by reference to protocol analyses in preliminary interview studies by the authors to indicate the degree that people put priorities on the self or the other in each scenario. Two items clearly related to giving priority to the self (for example, “Ignore the objection and get married”, “Explain your feelings to other and get married regardless of their opinion”), whereas the other two items clearly related to giving priority to others (for example, “Consider not getting married”, “Give up getting married”). Responses to the two items that prioritized others were reversed, and a composite score was obtained by calculating the mean of the four items as the “Self-assertion Score” for each story and for the Type of Other (Parent/Friend). Thus, higher Self-assertion Scores indicated a greater tendency to prioritize the self in each situation.

#### 2.2.2 Situational factors

Two key independent variables were the situational factors of Importance and Type of Other that were manipulated through the self–other dilemmas.

*Importance:* To manipulate the level of Importance, we created four dilemma stories: two stories representing a low level of importance (“TV” and “Lunch”) and two stories representing a high level of importance (“Job” and “Marriage”). The Low-Importance stories were conflicts about the person's own choices or preferences contrasted to the choices of others in small everyday situations (e.g., what TV program to watch: quiz show vs. sports game, and what to eat for lunch: Italian vs. Japanese). The High-Importance stories were related to important life decisions, such as accepting a desired offer to work for a company or getting married to someone the person really loves, but there are objections from the target person. (The full texts of all hypothetical self–other dilemma scenarios are provided in the Supplementary material 1).

*Type of Other*: Participants were asked to answer separately for cases in which the Type of Other varied. The four dilemmas were presented in relation to Parents and in relation to Friends. Type of Other (Parent vs. Friend) was manipulated within participants, such that all participants responded to each of the four dilemma scenarios twice, once for each target. This allowed for a comparison of a hierarchical relationship within the family and an egalitarian relationship outside of the family.

In addition, to conduct a manipulation check, a Challenge Score was constructed in which participants were asked one item to determine how challenging they found each dilemma. This item asked how much the participant would “Be at a loss, not knowing what to do” using a 6–point scale (from “Strongly disagree” to “Strongly agree”), and higher scores indicated that the participant found the dilemmas to be challenging to resolve.

Culture was represented by each country of origin that was coded as a categorical variable from 1 to 5, representing the U.S., China, Korea, Taiwan, and Japan, respectively.

Gender was explored as a potential variable contrasting female (0) with male (1) participants. Following preliminary analyses that revealed significant but small gender effects, this variable was used as a covariate in the central analyses.

Internal consistency of the total Self-assertion Score was examined separately for each country and Cronbach's alpha coefficients were acceptable across all cultural groups, ranging from 0.71 to 0.84 (U.S. = 0.84, China = 0.71, Korea = 0.73, Taiwan = 0.72, Japan = 0.80). To examine whether the same construct was measured across cultures, a multi-group confirmatory factor analysis (CFA) was conducted using a one-factor model including all 32 items. The model showed an acceptable level of fit to the data (RMSEA = 0.079), supporting configural invariance across countries.

## 3 Results

First, the importance of the dilemmas in the current sample was supported by examining the Challenge Scores. As shown in Table 1, mean scores on this item were significantly higher for the High-Importance scenarios than for the Low-Importance scenarios in all cultures (U.S.: *t* (140) = −8.62, *p* < 0.001, *d* = 1.49; China: *t* (178) = −8.20, *p* < 0.001, *d* = 1.08; Korea: *t* (240) = −13.17, *p* < 0.001, *d* = 1.09; Taiwan: *t* (185) = −13.97, *p* < 0.001, *d* = 1.15; Japan: *t* (411) = −18.69, *p* < 0.001, *d* = 1.18). These differences were observed for both Types of Others (Parent and Friend). Thus, as expected, a clear distinction in the level of Importance was found across all cultures, supporting the validity of this variable in the present study.

**Table 1:** Means (M) and standard deviations (SD) of challenge scores by culture.

**Importance**	**Type of Other**	**U.S**.		**China**		**Korea**		**Taiwan**		**Japan**	
		***M***	***SD***	***M***	***SD***	***M***	***SD***	***M***	***SD***	***M***	***SD***
Low	Parent	1.91	1.00	1.94	0.99	2.40	1.16	2.75	1.19	1.91	0.94
	Friend	2.06	1.18	2.08	1.04	2.85	1.31	2.95	1.31	2.37	1.26
	Total	1.98	1.05	2.01	0.97	2.63	1.15	2.85	1.20	2.14	1.00
High	Parent	3.29	1.56	2.91	1.56	3.92	1.36	4.42	1.36	3.58	1.55
	Friend	2.83	1.37	2.44	1.28	3.17	1.32	3.63	1.35	2.87	1.34
	Total	3.06	1.38	2.68	1.35	3.54	1.24	4.03	1.29	3.22	1.34

Upon closer examination of differences between Type of Other across cultures, conflicts with Friends were rated as more challenging than conflicts with Parents for the Low-Importance dilemmas (U.S.: *t* (141) = −3.11, *p* < 0.01, *d* = 0.58; China: *t* (179) = −2.96, *p* < 0.001, *d* = 0.63; Korea: *t* (243) = −7.91, *p* < 0.001, *d* = 0.88; Taiwan: *t* (186) = −3.58, *p* < 0.001, *d* = 0.74; Japan: *t* (412) = −9.91, *p* < 0.001, *d* = 0.95). However, this pattern was reversed for the High-importance dilemmas (U.S.: *t* (140) = 5.29, *p* < 0.001, *d* = 1.03; China: *t* (179) = 7.13, *p* < 0.001, *d* = 0.88; Korea: *t* (240) = 11.39, *p* < 0.001, *d* = 1.02; Taiwan: *t* (186) = 12.76, *p* < 0.001, *d* = 0.85; Japan: *t* (412) = 13.11, *p* < 0.001, *d* = 1.11). Thus, in all cultures, conflicts with parents were rated as more challenging in High-Importance situations, whereas conflicts with friends were rated as more challenging in Low-Importance situations.

Although there were slight variations in scores, the pattern of differences in Challenge Scores for Importance (Low/High) and Type of Other (Parent/Friend) was similar across cultures. These results therefore suggest that the dilemmas were largely comparable for college students in different cultures. In the following sections, we emphasize the results of the analyses of the Self-assertion Scores according to the situational factors of Importance and Type of Other. These results appear after brief explanations of the initial analyses that included all of the factors and the gender patterns. To facilitate interpretation, despite the potential issue of repeated testing, separate analyses and comparisons were conducted for each factor. Because these analyses were theory-driven and focused on a limited set of structured comparisons, we prioritized effect sizes and the consistency of observed patterns across analyses rather than applying a single global correction for all tests. Homogeneity of variance was examined using Levene's tests, and although these tests were significant for all dependent variables, the analyses were considered robust given the large sample size. In addition, because the repeated-measures factors had only two levels, the assumption of sphericity was not applicable.

### 3.1 The initial analysis of variance including all factors

First, the results of the mixed factor ANOVA (Importance:2 × Type of Other:2 × Gender:2 × Culture:5) indicated that all the main effects and many interactions were significant (as shown in Supplementary Table S1). The effect sizes of two situational factors (Importance and Type of Other) were largest (Partial η^2^ > 0.1), and those of the main effects of Culture and Gender and all the interactions were intermediate or small (0.05 < Partial η^2^ < 0.1), whereas other effects showed small sizes (Partial η^2^ < 0.05) except the interaction of Type of Other × Culture (Partial η^2^ = 0.091). Effect sizes (partial η^2^) were interpreted according to conventional benchmarks (Cohen, 1988). Thus, it was suggested that situational conditions of conflicts had larger effects for self-assertion in the dilemmas than Culture or Gender. However, it is noteworthy that the interaction between Culture and Type of Other showed a relatively large effect size.

### 3.2 Gender differences

Regarding gender differences, as shown in Table 2, males consistently scored higher than females in overall self-assertion across cultures. However, simple main effects of gender were significant only in Asian cultures (China: *t* (168) = 3.21, *p* < 0.001, *d* = 0.49; Korea: *t* (235) = 3.93, *p* < 0.001, *d* = 0.38; Taiwan: *t* (182) = 3.41, *p* < 0.001, *d* = 0.38; Japan: *t* (397) = 2.99, *p* < 0.01, *d* = 0.46), and not in the U.S. (n.s.). When examined by situational factors, no significant gender differences were found in the Low-Importance dilemmas across all Asian cultures, while significant gender differences with males scoring higher than females were observed in the High-Importance conditions.

**Table 2:** Means and standard deviations of self-assertion scores by gender in each culture.

**Gender**	**U.S**.		**China**		**Korea**		**Taiwan**		**Japan**	
	***M***	***SD***	***M***	***SD***	***M***	***SD***	***M***	***SD***	***M***	***SD***
Female	3.79	0.55	3.40	0.37	3.66	0.38	3.57	0.35	3.67	0.44
Male	3.86	0.48	3.59	0.43	3.86	0.39	3.77	0.42	3.82	0.51

Although the cultural variations in gender differences are intriguing, the effect sizes for both the main effect and the interaction of gender were small; therefore, gender was treated as a covariate (collapsed across gender) in the subsequent analyses of situational and cultural factors.

### 3.3 Effects of situational factors on self-assertion scores

**Figure 1:**
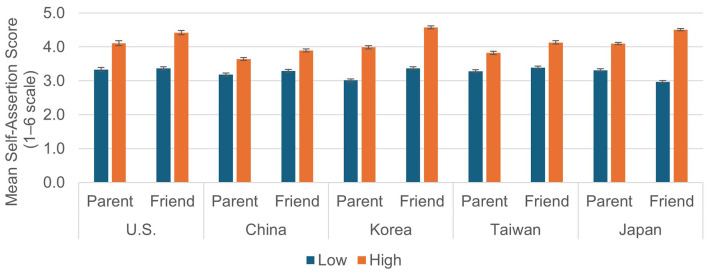
Mean self-assertion scores by importance (high vs. low) and type of other (parent vs. friend) across five cultures.

The mean Self-assertion Scores according to two situational factors are shown in Figure 1. A 2 (Importance) × 2 (Type of Other) ANOVA was conducted separately for each culture. The results were as follows: simple main effects of Importance were significant in all cultures, main effects of Type of Other were all significant except for Japan, and interactions were all significant in all cultures (statistical values are omitted for brevity). Because interactions were all significant in all cultures, in the following sections, differences between levels of each factor, as well as cultural differences, were examined.

#### 3.3.1 Effects of importance

**Figure 2:**
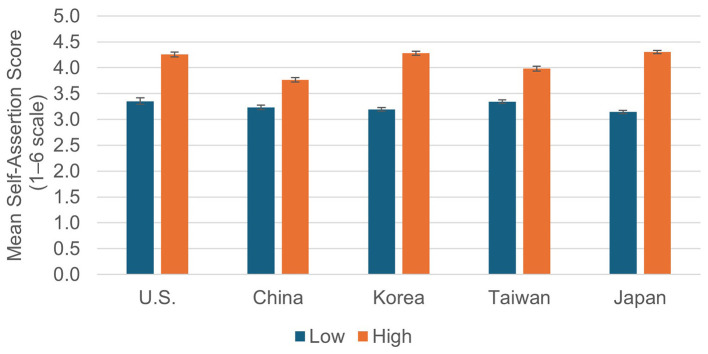
Means of self-assertion scores by culture and importance (high vs. low).

The mean scores by Importance (Low/High) are presented in Figure 2. As shown, the Self-assertion Score was higher for High-Importance dilemmas than for Low-Importance ones across all cultures. A 2 (Importance) × 5 (Culture) ANOVA revealed that the main effects of Importance (*F*_(1, 1118)_ = 964.61, *p* < 0.001, partial η^2^ = 0.463) and Culture (*F*_(4, 1118)_ = 12.49, *p* < 0.001, partial η^2^ = 0.043), as well as their interaction (*F*_(4, 1118)_ = 23.10, *p* < 0.001, partial η^2^ = 0.076), were all significant. Because the interaction was significant, simple main effects were examined, and these were significant in all cultures (U.S.: *F*_(1, 1118)_ = 142.79, *p* < 0.001, partial η^2^ = 0.113, China: *F*_(1, 1118)_ = 64.98, *p* < 0.001, partial η^2^ = 0.055, Korea: *F*_(1, 1118)_ = 357.96, *p* < 0.001, partial η^2^ = 0.244, Taiwan: *F*_(1, 1118)_ = 98.79, *p* < 0.001, partial η^2^ = 0.081, Japan: *F*_(1, 1118)_ = 722.79, *p* < 0.001, partial η^2^ = 0.393). Thus, participants were more self-assertive in situations concerning important events in every culture. In addition, the effect sizes were especially large (more than 0.08) in the U.S., Korea, and Japan, suggesting that the relation of situational importance to self-assertion was particularly pronounced in these cultures.

When focused on cultural differences, simple main effects of culture were significant both in Low and High-Importance dilemmas (Low: *F*_(4, 1118)_ = 6.12, *p* < 0.001, partial η^2^ =0.021, High: *F*_(4, 1118)_ = 11.48, *p* < 0.001, partial η^2^ = 0.089). The effect size was larger in High-Importance than in Low-Importance scenarios. According to the results of Bonferroni-adjusted multiple comparisons, in the Low-Importance dilemmas, only the differences between the U.S. and Japan, and between Taiwan and Japan were significant (*p*s < 0.001), whereas no other cultural differences were significant. Specifically, Japanese participants scored lower than those from Taiwan and the United States. In the High-Importance dilemmas, significant differences were found between the U.S. and China, the U.S. and Taiwan, China and Korea, China and Taiwan, Korea and Taiwan, and Taiwan and Japan (*p*s < 0.001). Overall, the mean scores indicated that self-assertion in High-Importance dilemmas was highest in Japan, followed by Korea and the U.S.; however, there were no significant differences among these three cultures. In contrast, participants from Taiwan and China showed significantly lower scores, with China scoring the lowest.

To summarize, situational importance was more strongly associated with self-assertion across cultures, and the magnitude of this association tended to be greater in Japan, Korea, and the U.S. than in Taiwan and China.

#### 3.3.2 Effects of type of other

**Figure 3:**
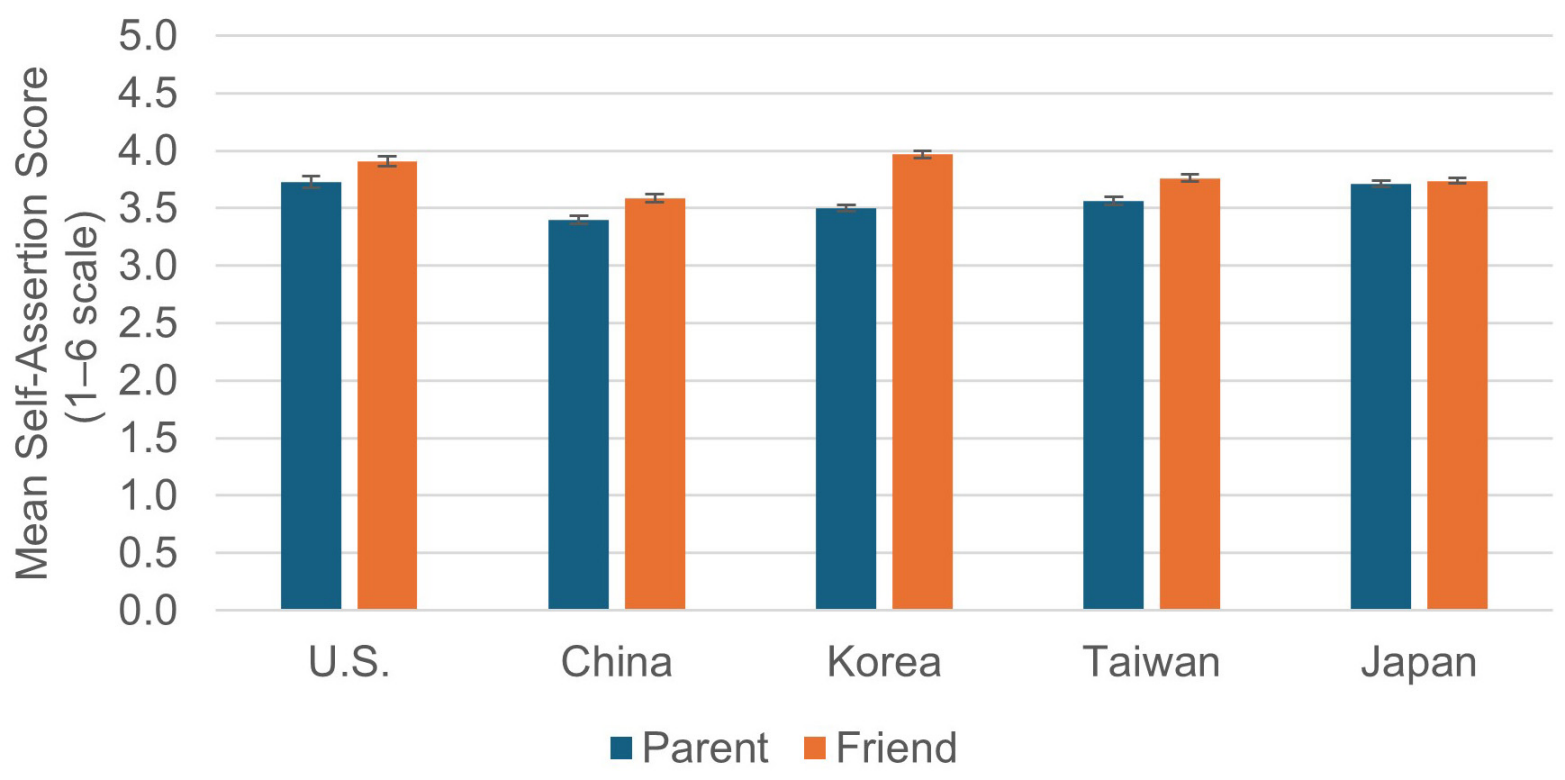
Means of self-assertion scores by culture and type of other (parent vs. friend).

Analyses were also conducted for the Type of Other. A 2 (Type of Other) × 5 (Culture) ANOVA was conducted for the data shown in Figure 3. The main effect of Type of Other (*F*_(1, 1118)_ = 216.93, *p* < 0.001, partial η^2^ = 0.162), Culture (*F*_(4, 1118)_ = 12.50, *p* < 0.001, partial η^2^ = 0.043) and their interaction (*F*_(4, 1118)_ = 36.54, *p* < 0.001, partial η^2^ = 0.116) were significant. Because the interaction was significant, simple main effects of the Type of Other were examined, and these were significant except in Japan (U.S.: *F*_(1, 1118)_ = 21.09, *p* < 0.001, partial η^2^ = 0.019, China:*F*_(1, 1118)_ = 29.56, *p* < 0.001, partial η^2^ = 0.026, Korea: *F*_(1, 1118)_ = 259.14, *p* < 0.001, partial η^2^ = 0.188, Taiwan: *F*_(1, 1118)_ = 35.50, *p* < 0.001, partial η^2^ = 0.031, Japan: n.s.). Thus, in all cultures except Japan, participants showed higher Self-assertion Scores for Friends than for Parents, and the effect size was particularly large in Korea.

When focusing on cultural differences, simple main effects of Culture were significant both in Parent and Friend conditions (Parent: *F*_(4, 1118)_ = 15.13, *p* < 0.001, partial η^2^ = 0.051, Friend: *F*_(4, 1118)_ = 20.37, *p* < 0.001, partial η^2^ = 0.068). The effect sizes were comparable across the two conditions and were of moderate magnitude. According to the results of Bonferroni-adjusted multiple comparisons in the Parent dilemmas, significant differences were found between the U.S. and China, the U.S. and Korea (*p*s < 0.001), China and Taiwan (*p* < 0.05), Korea and Japan (*p* < 0.05), and Taiwan and Japan (*p* < 0.05). Thus, in the Parent dilemmas, the United States and Japan showed the highest levels of self-assertion, China the lowest, and Korea and Taiwan were in between. In the Friend dilemmas, the difference between the U.S. and China (*p* < 0.001), the U.S. and Japan (*p* < 0.01), China and all the other cultures (*p*s < 0.01), Korea and Japan (*p* < 0.001), and Korea and Taiwan (*p* < 0.001) were significant. Thus, in the Friend dilemmas, Korea and the U.S. exhibited the highest scores, China the lowest, and Taiwan and Japan fell in between.

Taken together, the patterns of cultural differences in the Parent and Friend dilemmas indicate that the extent to which individuals differentiate between parents and friends in their self-assertion varied across cultures. In Korea, participants were consistently more self-assertive toward friends than toward parents. In contrast, in Japan there was no overall difference in self-assertion between parents and friends when averaged across importance levels. However, when situational importance was taken into account, Japanese participants showed slightly higher self-assertion toward parents than friends in Low-Importance situations (see Figure 1, the Importance × Type of Other interaction). In the United States and Taiwan, self-assertion levels were relatively similar across relationship types, whereas in China self-assertion was generally low regardless of the target person. These findings suggest that the extent to which individuals differentiate between parents and friends in their self-assertion is itself culturally patterned rather than universal.

Finally, cultural differences were analyzed in the composite Self-assertion Scores, with the effects of the other factors collapsed. The means and standard deviations of Self-assertion Scores were as follows: the U.S. (*M* = 3.82, *SD* = 0.53), China (*M* = 3.49, *SD* = 0.41), Korea (*M* = 3.73, *SD* = 0.39), Taiwan (*M* = 3.66, *SD* = 0.39), and Japan (*M* = 3.72, *SD* = 0.47). The results of the one–way ANOVA with Culture as the independent variable were significant (*F*_(4, 1122)_ = 12.50, *p* < 0.001, partial η^2^ = 0.043) and post-hoc multiple comparisons using the Bonferroni adjustment revealed that China scored significantly lower than all other countries (Japan, Korea, Taiwan, and the U.S., *p*s < 0.01). Thus, when averaged across all conditions, the overall level of self-assertion was relatively similar among Korea, Taiwan, Japan and the U.S., whereas China showed lower scores than the other cultures.

## 4 Discussion

The present study examined situational and cultural variations in self-assertion among young adults in five cultural contexts. Using hypothetical self–other dilemma situations that varied by the importance of the issue and the type of relationship partner, this research sought to clarify how young people negotiate autonomy and relatedness across different social contexts. Overall, the findings revealed both commonalities across cultures and culture-specific patterns of self-assertion, supporting the view that autonomy and relatedness coexist as dynamic and context-dependent processes in human development. Taken together, the results showed strong support for the situational predictions across cultures, whereas support for the cultural predictions was partial and characterized by more differentiated patterns, particularly within East Asia.

### 4.1 Commonalities of context-sensitive self-assertion by situational importance

Across all cultural groups, participants were more self-assertive in situations involving highly important issues than in those of low importance. This robust main effect of situational importance confirms that when personal stakes are high, individuals are more likely to prioritize their own needs regardless of cultural background. More specifically, this study supported the hypothesis that when the issues concerned important life matters such as marriage or career choice, participants tended to maintain their own preferences and assert their desires. In contrast, when the matters were more peripheral for them, such as what to watch on TV or where to go out for lunch, they tended to yield to others' requests. These negotiation patterns between self and others were consistently observed across the five cultures in this study.

This pattern is consistent with our previous findings with Japanese participants (e.g., Hirai, 2000, 2006), which formed the foundation for the present study. In addition, (Sugimura et al. 2009) also examined Japanese adolescents' responses to hypothetical disagreements with parents and found that negotiation and self-assertion were more likely to occur in more serious situations. Together with earlier work, the current cross-cultural evidence suggests that context-sensitive self-assertion is not a uniquely Japanese pattern but reflects a more general tendency. The results of this situational effect of importance indicate that this modulation of self-assertion is not a culture-bound phenomenon but was a broadly consistent tendency for the countries represented in this study.

It is also important to note that when self–other dilemmas occur, people do not always prioritize the self, even when the issue is highly important. Rather, it is a matter of degree and balance. Our previous interview study revealed, through participants' narratives, that because significant others matter deeply to them, individuals attempt to explain or persuade in order to be understood, rather than simply insisting on their own views. People do not make all-or-nothing decisions; instead, they seek a balance in daily interactions, asserting themselves to others when the matter is personally meaningful (Hirai, 2000, 2006, 2017; Hirai and Takahashi, 2000).

This view is also consistent with recent research on parent–adolescent conflict, which emphasizes that conflicts are organized around specific everyday issues and vary in their psychological meaning depending on the salience of those issues. For example, (Mokhtarnia et al. 2023) showed that disagreements between parents and adolescents cluster around concrete domains of daily life (e.g., autonomy, leisure, or values), and that conflict intensity reflects issue-specific importance rather than a generalized tendency toward opposition or compliance. From this perspective, self-assertion can be understood as a situationally calibrated response within concrete conflict contexts, through which individuals regulate both autonomy expression and relational harmony, rather than as a fixed trait or a zero-sum trade-off between autonomy and relatedness. In the present study, this pattern was reflected in systematic differences in self-assertion between high and low importance situations. Thus, young adults across cultures calibrated their self-assertion in ways that balanced self and other according to situational demands.

This situational modulation of self-assertion is also consistent with research on cultural differences in conflict management strategies, which distinguishes patterns such as avoidance, accommodation, and negotiation (e.g., Rahim, 2002; Ting–Toomey, 2005). Rather than reflecting fixed cultural preferences for particular strategies, prior work suggests that individuals flexibly adjust their level of assertiveness depending on situational demands and relational considerations. The present findings extend this literature by showing that self-assertion varies systematically as a function of issue importance, highlighting negotiation as a context-sensitive process rather than a stable cultural trait. From this perspective, self-assertion in the present study can be understood as one form of negotiation-oriented conflict management, whose expression varies flexibly across situations and relationships rather than reflecting a fixed cultural preference.

Regardless of any demands of significant others or any kind of cultural expectations, autonomous decisions regarding what was important to themselves were evident through self-assertion. The ability to manage and negotiate social conflicts by asserting personal desires while respecting the positions of others is a critical part of healthy development in many cultures. Related research further indicates that how conflicts are negotiated within close relationships is linked to adolescents' emotional functioning, including social withdrawal and loneliness (Kong et al., 2024). The self as an active agent involves the capacity to decide, express, and sometimes negotiate with others. Identity studies have suggested that experiences involving these kinds of dialectical and dynamic tensions between self and other or autonomy and relatedness are important processes of identity development particularly during adolescence and young adulthood (e.g., Lichtwarck–Aschoff et al., 2008, 2009). Thus, identity or self has a notable relational component. Through these clashes, people learn about and develop their own identities throughout their lives. Such forms of self–other negotiation may not be limited to adolescence and young adulthood, but may instead characterize interpersonal development across the lifespan.

In summary, as this study has partially demonstrated, people strive to be both autonomous and relational and this tendency transcends cultural boundaries. These findings are consistent with the autonomous-related self that (Kagitçibaşi 2005, 2007, 2013) has emphasized. Furthermore, the present findings also align with Self-Determination Theory (Ryan and Deci, 2017; Vansteenkiste et al., 2020), which posits that the need for autonomy is universally salient, even though its expression is shaped by situational and cultural conditions.

### 4.2 Cultural commonalities and differences in self-assertion toward parents and friends

This situational modulation of self-assertion was further adjusted according to the type of relationship, that is, whether the counterpart was a parent or a friend. When the factor of situational importance was collapsed, participants were generally more self–assertive toward friends than toward parents in most cultures, except in Japan. In Japan, the difference in the degree of self-assertion between Low- and High-Importance situations was especially pronounced in the Friend condition. Japanese young adults were less self-assertive (the lowest Self-assertion Score) for the Low-Importance Friend dilemma, while they asserted themselves to a similar degree as those from other cultures in dilemmas involving important issues with friends. Thus, even Japanese youth are not always unassertive toward their friends; rather, they do assert themselves when the issue is important. In sum, regardless of culture, self-assertion was modulated according to the type of other.

The pattern to be more self-assertive toward friends than toward parents is thought to reflect the different normative expectations associated with hierarchical vs. egalitarian or reciprocal relationships. In general, the parent–child relationship is characterized by protection and dependence when the child is young and is therefore not an equal relationship. In contrast, friendships are relationships between peers of equal status. An egalitarian and intimate relationship is one in which both parties trust each other and respect one another's individuality (e.g., Bukowski et al., 2020; Lerner, 1989), hence, both the self and the other are expected to be able to express themselves assertively.

The particularly large effect size in Korea suggests that distinctions between filial and peer relationships are especially pronounced in societies where Confucian values of parental authority and filial duty remain salient (e.g., Choi and Kim, 2006). Korean young adults were more self-assertive to friends, but not to parents. The high scores of Korean young adults in the friend condition might indicate an ongoing cultural negotiation between traditional deference to authority and the strong agency fostered in a highly competitive, achievement-oriented society.

On the other hand, the tendency among Japanese young adults not to assert themselves in Low-Importance dilemmas with friends may reflect culturally embedded norms emphasizing interpersonal harmony and the avoidance of unnecessary conflict. In Japanese culture, maintaining smooth and trusting relationships often takes precedence over expressing one's own preferences, especially when the issue is trivial. This tendency is closely related to the cultural norm of *enryo* (restraint or considerate hesitation), which involves withholding one's opinions or desires out of respect for others' feelings to avoid imposing on them. In low-importance situations, exercising *enryo* is regarded as a sign of maturity and social sensitivity rather than weakness. It has also been pointed out that Japanese people have peculiar interpersonal anxiety, sometimes it is called *taijin kyofu* (social phobia), as a Japanese culture-specific syndrome (e.g., Tanaka–Matsumi, 1979). In contrast, such restraint tends to operate less strongly within the family. Because family ties are regarded as secure and enduring, individuals often feel less need to exercise *enryo* or *taijin kyofu* and may express their opinions more directly, even when disagreement occurs. This can be understood in light of the Japanese distinction between *uchi* (inside) and *soto* (outside), which defines relational boundaries in social life. Within *uchi* relationships, such as those among family members, emotional exchange, including disagreement or confrontation, are more permissible, as the bond is assumed to be stable and unconditional. By contrast, *soto* relationships, including friendships, require greater social sensitivity and self-restraint to maintain harmony. Thus, *enryo* is more likely to be activated in *soto* contexts, whereas its influence diminishes within the *uchi* sphere. The relative lack of *enryo* toward parents may allow individuals to assert themselves more freely in family contexts even in an issue that is not-highly important.

### 4.3 Theoretical implications for cultural psychology

Up to this point, we have focused on culturally shared tendencies in situational negotiation, which were largely consistent with our initial expectations, as well as on culture-specific relational differences toward parents and friends that revealed more differentiated patterns, particularly as they interacted with situational importance in Japan and Korea. Together, these findings suggest that East Asian cultures cannot be treated as homogeneous.

When we examined self-assertion across situational factors, young adults in the U.S. showed the highest mean scores, although they did not differ significantly from those in Korea, Taiwan, or Japan. China, however, scored significantly lower than the other four cultures. When examined by situational Importance, in Low-Importance dilemmas, self-assertion was higher in the U.S. and Taiwan and relatively lower in Japan. In High-Importance dilemmas, self-assertion was high in Japan, Korea, and the U.S., whereas it remained relatively low in China. When examined by Type of Other, in dilemmas involving parents, self-assertion was high in the U.S. and Japan and lowest in China. In dilemmas involving friends, scores were high in the U.S. and Korea, with China again showing the lowest levels. Although the specific patterns of cultural differences varied across the situational factors examined in this study, a general trend emerged in which participants in the United States tended to be consistently more self-assertive, whereas those in China were often less so.

When self-assertion is conceptualized as a self-oriented characteristic, this overall pattern aligns with long-standing theoretical accounts contrasting Western and Eastern cultural emphases. However, as seen in Korea and Japan, strong self-assertion also emerged under certain situational conditions even within East Asian cultural contexts. It is noteworthy that culture played a considerably smaller role for both situational importance and type of other. These results underscore the importance of considering both cultural norms and situational contingencies when interpreting cross-cultural differences in self-assertive behavior. Moreover, they highlight that East Asian cultures themselves are not monolithic but exhibit meaningful diversity in patterns of self-assertion.

In recent cultural psychology, researchers have moved beyond the traditional dichotomy of the “independent self” in Western contexts and the “interdependent self” in Eastern contexts, seeking more diverse and dynamic frameworks of cultural mindsets. For example, (Kitayama and Salvador 2024), in their comprehensive review in *Annual Review of Psychology*, conceptualized culture as an eco-cultural complex, in which ecological, geographical, historical, and institutional factors interact dynamically. They proposed that cultural differences should not be viewed as static structures but as co-evolutionary processes of environmental adaptation and social institutions. Within this framework, distinctive forms of interdependence have been identified not only in East–West comparisons but also in diverse regions such as Latin America, South Asia, and the Arab world. However, even within this model, East Asian cultures are often treated as a single category. Similarly, (Kitayama et al. 2022) extended the study of the culturally constructed self to the neuroscientific and genetic levels, proposing that the self is a collective construction shared within culture. From this perspective, culture is not merely a system of psychological values, but a social blueprint inscribed in brain functioning and genetic sensitivity. These recent theoretical developments point toward reconceptualizing culture and the culturally shaped self not as fixed categories but as multilayered, dynamic processes that connect biological, individual, and societal levels.

Taken together, the present findings are consistent with the view that concerns related to autonomy and relatedness are flexibly coordinated across cultures, rather than operating as mutually exclusive dimensions, and with recent developments in cultural psychology that move beyond static East–West dichotomies. From this cultural perspective, culture should not be understood as a simple dimension on which societies can be placed, but rather as a dynamic system in which individuals construct the self flexibly in response to varying social contexts (Kitayama and Salvador, 2024). From a developmental perspective, this interpretation also aligns with contemporary accounts that conceptualize autonomy as a relational and context-dependent process rather than a fixed individual trait (Smetana et al., 2024). Accordingly, higher levels of self-assertion in the present study should not be interpreted as reflecting greater autonomy *per se*, nor should lower self-assertion be equated with greater relatedness.

### 4.4 Gender differences

This study was limited in what could be concluded about gender patterns, as it did not aim to provide a detailed examination of gender differences in self-assertion. In the present data, males tended to score higher than females in overall self-assertion across cultures; however, these differences were small in magnitude and not consistently observed across cultural and situational contexts. Specifically, significant gender differences were found primarily in High-Importance dilemmas within Asian cultures, whereas no significant gender differences were observed in the United States. Given the small effect sizes for both the main effect and interactions involving gender, gender was treated as a covariate with subsequent analyses collapsed across gender to focus on situational and cultural factors. At the same time, the absence of robust gender effects in the present study does not imply that gender is irrelevant to the development or expression of self-assertion. Rather, gender-related patterns of self-assertion are likely to be shaped by broader cultural and institutional contexts.

Gendered behavioral tendencies such as self-assertion may reflect broadly shared patterns; however, their expression and social meaning are likely shaped by culturally specific norms and institutional conditions related to gender equality, suggesting the potential importance of considering the intersection of gender and culture in future research. This perspective is consistent with research emphasizing that gender-related behaviors are embedded in cultural systems of meaning and socialization (Best and Puzio, 2019), and that gender differences are not uniform across societies but vary depending on broader cultural and normative contexts (Guimond et al., 2010).

With regard to gender, a well-known and well-referenced index of cross-national variations is the Global Gender Gap ranking by the World Economic Forum. According to the latest Global Gender Gap Report 2025, the five countries in this study differ considerably in their rankings: the U.S. ranked 43rd (score = 0.754), South Korea 99th (0.689), Taiwan (Chinese Taipei) 114th (0.670), Japan 118th (0.664), and China 130th (0.667) among 148 economies (World Economic Forum, 2025). This indicates that the degree of gender equality varies markedly even among major industrialized and East Asian societies.

Future research should more directly examine gender-by-culture interactions using designs specifically powered and theorized to address such questions. In particular, further studies should investigate how autonomy–related behaviors, such as self-assertion, are shaped by gendered socialization processes operating within specific cultural contexts and situational conditions.

### 4.5 Limitations and future directions

In addition to the issues regarding gender differences discussed above, several limitations of the present study point to important directions for future research.

First, the present study employed hypothetical dilemmas, and it remains uncertain whether people would behave in similar ways in real–life situations. Moreover, the psychological meaning of the scenarios may differ across cultural contexts. For example, decisions such as choosing a marriage partner may be understood primarily as an individual choice in some cultures, whereas they may be viewed as a family-negotiated process in others. Such differences in interpretation could influence how self-assertion is expressed and should be considered when interpreting cross-cultural differences. Future studies could incorporate behavioral observations, ecological assessments, or qualitative approaches to better capture culturally grounded meanings of self-assertion.

Second, the scenarios and situational factors used in this study were limited in scope. Other contextual elements, for example power balance, emotional intensity, or the presence of third parties, may also influence self-assertion and deserve further examination.

Third, the study did not investigate associations with widely used psychological measures, such as indicators of psychological well-being or cultural self-construal. Future research should explore how self-assertion scores relate to such established constructs to better situate the findings within broader psychological frameworks.

Finally, because the participants were university students, the sample cannot be considered representative of each culture as a whole. Including more diverse age groups and occupational backgrounds would provide a more comprehensive understanding of cultural and developmental variations in self-assertion.

## 5 Conclusion

In conclusion, the present study provides cross-cultural evidence that self-assertion, conceptualized as a situational behavioral expression in self–other dilemmas, is both consistent in some ways across cultural contexts and flexibly shaped by situational and relational conditions. While the tendency to assert oneself in highly important situations appears to be a broadly shared characteristic across multiple cultural contexts, the relational and cultural contours of this tendency differ across societies. These results underscore the importance of examining autonomy and relatedness not as fixed cultural tendencies or opposing dimensions, but as dynamically interwoven aspects of self-development that are jointly negotiated through context–sensitive patterns of self-assertion within specific sociocultural environments.

## Data Availability

The raw data supporting the conclusions of this article will be made available by the authors, without undue reservation.
