# Supplementary material for: Situational self-assertion in self–other dilemmas among young adults in five cultures: negotiations between self and others
Source: Front Psychol. 2026 Mar 3;17:1766805. doi: 10.3389/fpsyg.2026.1766805 (PMC12992035; doi:10.3389/fpsyg.2026.1766805)
Supplement: Supplementary file 1 [file Data_Sheet_1.docx]

**Supplementary Material 1.**

**Full Texts of the Self–Other Hypothetical Dilemma Scenarios**

In each scenario, participants were asked to imagine the target person (X) as either a parent or a friend.

TV (Low Importance)

You are watching TV with X after a meal. You want to watch a quiz show, but X says he/she wants to watch sports.

Lunch (Low Importance)

You are going to have lunch with X. You were thinking of an Italian restaurant, but X insists on a Japanese restaurant.

Job (High Importance)

After job hunting, you have finally received an offer from a company. This is the company that you really want to work for, but when you told X about it, X objected to your accepting the offer.

Marriage (High Importance)

You have a boyfriend /girlfriend. Gradually, you come to think that you want to marry this person. However, when you told X about it, X objected to your getting married.
